# Quantitative Assessment of Antimicrobial Activity of PLGA Films Loaded with 4-Hexylresorcinol

**DOI:** 10.3390/jfb9010004

**Published:** 2018-01-11

**Authors:** Michael Kemme, Regina Heinzel-Wieland

**Affiliations:** Department of Chemical Engineering and Biotechnology, Hochschule Darmstadt, University of Applied Sciences, Stephanstrasse 7, 64295 Darmstadt, Germany; regina.heinzel-wieland@h-da.de

**Keywords:** PLGA, 4-hexylresorcinol, biomaterial, antimicrobial, agar overlay assay, MIC

## Abstract

Profound screening and evaluation methods for biocide-releasing polymer films are crucial for predicting applicability and therapeutic outcome of these drug delivery systems. For this purpose, we developed an agar overlay assay embedding biopolymer composite films in a seeded microbial lawn. By combining this approach with model-dependent analysis for agar diffusion, antimicrobial potency of the entrapped drug can be calculated in terms of minimum inhibitory concentrations (MICs). Thus, the topical antiseptic 4-hexylresorcinol (4-HR) was incorporated into poly(lactic-co-glycolic acid) (PLGA) films at different loadings up to 3.7 mg/cm^2^ surface area through a solvent casting technique. The antimicrobial activity of 4-HR released from these composite films was assessed against a panel of Gram-negative and Gram–positive bacteria, yeasts and filamentous fungi by the proposed assay. All the microbial strains tested were susceptible to PLGA-4-HR films with MIC values down to 0.4% (*w*/*w*). The presented approach serves as a reliable method in screening and quantifying the antimicrobial activity of polymer composite films. Moreover, 4-HR-loaded PLGA films are a promising biomaterial that may find future application in the biomedical and packaging sector.

## 1. Introduction

Microbial contamination of polymeric biomaterials constitutes a major challenge in the fields of consumer protection, food safety, and human healthcare around the world [1]. For medical devices and drug delivery systems, microbial attachment adversely affects the functionality and limits the lifetime of biomaterials, and device-associated nosocomial infections represent a common and substantial complication in healthcare units [2]. Similarly for food processing and packaging materials, microbial accumulation on surfaces has a serious impact on quality and safety of food products, as well as consumer health [3]. Functionalisation of polymers with antimicrobial drugs or biocides has aroused considerable interest for the development of contact-active or drug-releasing biomaterials with resistance to microbial colonisation and spreading of pathogenic microorganisms [4,5].

Among all the biomaterials, the aliphatic polyester poly(lactic-co-glycolic acid) (PLGA) has found broad acceptance for controlled drug release in humans via microparticles or implants approved by the U.S. Food and Drug Administration (FDA) and the European Medicines Agency (EMA), respectively [6,7]. As a biodegradable polymer with excellent biocompatibility, low systemic toxicity, and tunable mechanical properties, PLGA is widely used as drug carrier to protect active molecules from harsh environments and to improve their delivery and uptake via oral or parenteral administration [8]. The complete release of incorporated molecules is achieved through a collective process of drug diffusion, ester bond hydrolysis, and bulk erosion of the polymer matrix [9]. PLGA-based drug delivery systems can be easily formed into any shape and size, such as particles, fibres, scaffolds, films, and coatings, and they can be used to incorporate and deliver a wide range of synthetic and natural drugs [10,11]. Numerous hydrophobic agents have been incorporated into PLGA matrices with proven antimicrobial efficiency, for example, chlorhexidine [12], essential oils from clove [13] or thyme [14], the flavonoids quercetin [15] and rutin [16], the monoterpenes carvone [17] and carvacrol [18], the phenylpropanoids anethole [17], cinnamaldehyde [19], and eugenol [19], a plant extract from guabiroba [20], and the steroid-like antibiotic fusidic acid [21].

In recent years, a number of pharmacological studies have been made on alkylresorcinols, which comprise a family of hydrophobic, non-isoprenoid phenolic lipids naturally synthesised by higher plants [22]. The synthetic derivative 4-hexylresorcinol (4-HR, Table 1) is reported to have anesthetic, antiseptic, and antihelmintic properties and is widely used as topical drug in antibacterial oral gargles and throat lozenges [23]. The 4-HR inhibits human transglutaminase 2, the NF-κB pathway, and intracellular calcium oscillations, thus making it a promising anti-cancer agent showing synergistic effects with cisplatin [24]. Suppression of NF-κB phosphorylation and inhibition of foreign-body giant cell formation is associated with anti-inflammatory effects of 4-HR [25]. Additionally, 4-HR has been described as a potent inhibitor of tyrosinase and polyphenol oxidase, thereby inducing antioxidant effects that are utilised in anti-ageing or skin lightning cosmetics, as well as in food processing aids to avoid melanosis in shrimps [23]. Based on a long history of human safe use, 4-HR is FDA-listed as active ingredient for over-the-counter products and is approved as a food additive (E 586) in the European Union [26].

Antibacterial properties of 4-HR towards single test strains have been reported infrequently since 1924, but quantitative data on 4-HR antisepsis have not been thoroughly determined [27]. Furthermore, systematic studies on antimicrobial susceptibility profiles or proof-of-concept experiments for drug delivery systems based on polymer-incorporated 4-HR are not yet available. Potency of leachable biocide-containing polymers is usually tested in vitro by direct-contact antimicrobial assays, such as Kirby–Bauer agar disc-diffusion or soft-agar overlay techniques [28,29,30]. In these solid diffusion tests, contact between the antimicrobial agent and the inoculated media takes place on the sample surface, thus simulating in vivo applications. The effectiveness of the antimicrobial drug can be assessed by its ability to suppress microbial growth, quantitatively described by the lowest concentration of the drug at which the test strain is completely inhibited (minimum inhibitory concentration, MIC) [31]. Standard agar disc-diffusion assays, however, provide only semi-quantitative information recording diameters of inhibition growth zones around the polymer sample, as it is impossible to exactly determine the free drug amount in the agar medium [32].

This paper investigates the physical and biocidal properties of glass-supported PLGA films loaded with different percentages of 4-HR, prepared by a solvent-casting technique. To explore its potential as an antimicrobial coating in packaging or biomolecular applications, we developed a rapid, effective, and reproducible assay for antimicrobial activity testing of 4-HR-loaded PLGA films. Using a modified seeded lawn agar overlay technique in combination with two different diffusion models for data analysis, this assay allows a fast and reliable screening of different indicator strains whose antimicrobial susceptibility is going to be evaluated quantitatively in terms of MIC values.

## 2. Results and Discussions

### 2.1. Effect of 4-HR on PLGA Film Properties

Supported PLGA-4-HR composite films were fabricated by solvent casting, leaving translucent white polymer films on the coverslips as glass substrate, which serves as a substitute for a coated device. Since 4-HR is very poorly soluble in water [26], dichloromethane was used as organic solvent allowing the production of thin films with high 4-HR concentrations up to 50% (*w*/*w*). The high miscibility of 4-HR with PLGA may be attributed to the presence of phenol groups that can form hydrogen bonds with ester bonds in the backbone of PLGA [33]. Vigorous stirring of the solvent should assist in uniform dispersion of 4-HR into the PLGA matrix. All films were homogeneous and smooth at macroscopic level and easily handled, except for samples with the highest proportion of 4-HR, due to their marked stickiness.

The effect of 4-HR on physical properties of PLGA films was characterised by determining data of film thickness and density, respectively (Table 2). These parameters are important for the application of PLGA composite films and the reliability of measured antimicrobial properties with regard to mechanical, barrier, and transport properties [34]. All thickness values ranged from 64 µm to 92 µm and did not significantly change when 4-HR content increased from 0 to 0.5 mg/mg PLGA. This result suggested that 4-HR could not expand the film structure through enlargement of the molecular volume inside the PLGA network. Moreover, the thickness of PLGA composites produced in this work was similar to neat PLGA films (85–100 µm) made by a solvent-casting technique under comparable conditions [35]. The mean standard deviation within the film specimen was about 15% of the average thickness indicating minor batch-to-batch variations, which are characteristic for the non-continuous solvent-casting process [8].

The thickness and mass of the films were used to calculate their density (Equation (1)). The addition of a small quantity of 4-HR (up to 6% (*w*/*w*) relative to PLGA) does not significantly change the density of composite films compared to neat PLGA specimen. However, a significant increase in the density of PLGA films was evidenced at higher values of 4-HR from 15–50% (*w*/*w*). This finding can be related to strong interactions between hydrophobic blocks in polymer chains and the hydrophobic drug. Therefore, it is concluded that an excess of 4-HR was able to cover PLGA molecules during the solvent-casting process increasing polymer chain flexibility in the manner of a surfactant, at the same time allowing a dense packaging of the composite molecules with a higher film density. According to product specification, pure PLGA powder should have a bulk density of 1.25 g/cm^3^ [36]. The considerably lower density values in our study may well be associated with the plasticizer effect caused by the absorption of water by the PLGA films [37].

### 2.2. Antimicrobial Performance of 4-HR-Loaded PLGA Films

To the best of our knowledge, the antimicrobial potential of 4-HR entrapped in a biopolymer matrix has not been explored. Thus, the in vitro antimicrobial activity of PLGA-based films containing different 4-HR concentrations in their coatings was screened against four Gram-positive bacteria, four Gram-negative bacteria, two yeasts, and two filamentous fungi (Table 3). The test organisms were selected for safety reasons because all are well-characterised laboratory strains that act as counterparts of pathogenic organisms relevant to healthcare-associated infections, personal care applications, and food spoilage [38,39]. The test method combines the agar disc diffusion with a homogeneous lawn of microbial cells within a thin layer of agar across the surface of the plate to ensure a uniform microbial distribution (Figure 2). High seeding densities of microbial cells from 10^5^ CFU/mL to 10^6^ CFU/mL were applied to simulate a worst case scenario of infection. This agar overlay assay is based on the measurement of clearing zones free of visible microbial colonies caused by growth inhibition of the microbial lawn in direct contact with the active agent. The hydrophobic nature of 4-HR bears a challenge for microbial inhibition because delivering 4-HR molecules to microbial cells in aqueous media is hampered. However, recent studies on 4-HR delivery systems using Fourier-transform infrared spectroscopy displayed broad absorption peaks that can be attributed to hydrogen bonding between 4-HR, physically adsorbed water molecules, and hydrophilic matrices such as agar for microbiological work [40,41]. Taken together, these findings and the observed microbial inhibition indicate that a portion of 4-HR was released from the PLGA matrix and diffused into the agar layer in a radial manner retarding the development of microbial cells in the agar overlay. The diameter of the inhibition zone reflects the susceptibility of the test organism.

**Figure 1:**
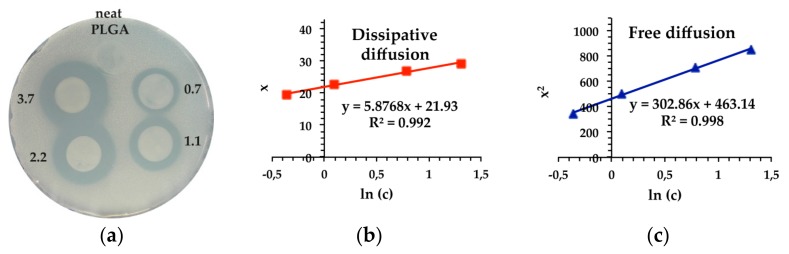
Agar overlay assay of PLGA–4-HR film discs against *Staphylococcus carnosus* as test organism. Diameter of inhibition zones x (mm, red squares) and their squared values x^2^ (blue triangles) are plotted against the logarithm of 4-HR concentration together with linear fits. (**a**) Representative inhibition halos with 4-HR concentrations from 0.7 mg/cm^2^ to 3.7 mg/cm^2^ PLGA surface area; (**b**) Plot for dissipative diffusion model (Equation (3)); (**c**) Plot for free diffusion model (Equation (2)).

The PLGA films loaded with 4-HR ranging in concentrations from 0.1 mg to 0.5 mg 4-HR/mg PLGA (i.e., 0.7–3.7 mg/cm^2^) showed good antimicrobial activity in a concentration-dependent manner compared to neat PLGA as control film, which did not display any inhibitory effect (Figure 1a). The average area of the regularly formed zones increased steadily by increasing the concentration of incorporated 4-HR to 3.7 mg/cm^2^.

However, more precise data on antimicrobial properties were obtained through determination of bacteriostatic concentrations reported as MIC values. By testing varying concentrations of the antimicrobial drug in the agar overlay assay, the minimal amount of 4-HR required for a visible decrease in microbial growth can be calculated from new algorithms for diffusion proposed by Bonev et al. [32]. The development of clearing zones depends on the nature of diffusion of 4-HR through the agar overlay characterised by two diffusion concepts. The free diffusion model is based on the assumption that the antimicrobial drug diffuses freely in the solid matrix. By contrast, the dissipative diffusion model takes account of drug loss through interactions with the agar matrix, aggregation of drug molecules, or drug inactivation to some extent.

Both models were used to calculate MIC values for 4-HR (Figure 1b,c and Table 3) because the validity of each algorithm can depend on the performance of 4-HR which has still not been fully resolved. Though plots of both the free and dissipative diffusion model indicated linearity, the corresponding *R*^2^ values of linear regression were higher for the free diffusion model. Thus, diffusion of 4-HR through the solid agar overlay can be considered as a free diffusion process which yields the best suit model for the determination of MICs.

Using the modified agar overlay assay, 4-HR-loaded PLGA films showed antimicrobial activity against all of the tested bacteria, yeasts, and filamentous fungi, although presenting different levels of growth inhibition (Table 3). The MIC values obtained from the free diffusion model ranged from 3.0% to 28.5% (*w*/*w*) and from 0.4% to 12.0% (*w*/*w*) for the dissipative diffusion model, respectively. Gram-positive *Staphylococcus carnosus* with a MIC down to 0.4% (*w*/*w*) was the most sensitive to PLGA–4-HR composite films, whereas Gram-negative *Pseudomonas putida* was the least sensitive strain. This 4-HR resistance can be explained through degradation of the antimicrobial agent, since the catabolic potential of *P. putida* against resorcinols is well documented [42]. All other tested microbial strains displayed similar degrees of susceptibility with a median MIC of 7.5% (*w*/*w*), independently of the Gram status or the fungal kingdom.

A previous investigation of the antimicrobial activity of 4-HR solutions yielded MIC values ranging from 0.3 µg/mL to 50 µg/mL against various bacteria and fungi relevant to personal care applications [23]. Due to differences in the test methods, it is impossible to compare this data with the effectiveness of PLGA-incorporated 4-HR concentrations in our study. Recently, the antimicrobial activity of silk sutures with 12% (*w*/*w*) 4-HR was tested using the Kirby–Bauer agar diffusion technique [43]. The data clearly showed that drug-loaded silk discs had a less significant effect on growth of Gram-negative *E. coli* compared with five Gram-positive pathogens. This can be attributed to a slow release of 4-HR which strongly adsorbs to silk fibroin, thus lowering the active drug concentration necessary to penetrate the lipopolysaccharides in the outer membrane of *E. coli*. The authors have only addressed the antibacterial activity in terms of clearing zone diameters, with no specific reference to MIC values. Therefore, our study gives an indication of the appropriate 4-HR amount in PLGA films required to inhibit a broad spectrum of microbial strains. Finally, the demonstration of the antimicrobial activity of PLGA-embedded 4-HR against Gram-positive bacteria, Gram-negative bacteria, yeasts, and filamentous fungi is an indication that thin PLGA-4-HR composite films can be used in the treatment of microbial pathogens irrespectively of their cell wall compositions.

The mode of action of 4-HR on microbial cells should be similar to other phenolic lipids, which interact with biological membranes and intracellular proteins [44]. Due to its amphiphilic structure, 4-HR is able to migrate through cell membranes leading to the breakdown of membrane integrity and increasing membrane permeability, which disrupts many vital processes such as energy production and nutrient transport. In addition, 4-HR can affect protein structure and activity to destroy metabolic pathways. Alkylresorcinols like 4-HR appear to act upon multiple targets, thus lowering the tendency to acquire pathogen resistance compared with conventional antibiotics [45].

While this study demonstrates the specific efficacy of PLGA–4-HR composite films, the agar overlay assay used herein is highly versatile and easy amendable to the inclusion of alternative antimicrobials or extension to other film-based drug delivery systems. Over the last decade, a wide range of polymeric antimicrobial carriers (e.g., bioceramics [46], biofoams [47], metal coatings [48]) has been explored tentatively, thus offering new application possibilities for the proposed assay.

## 3. Materials and Methods

### 3.1. Materials

Alky ester end-capped PLGA RESOMER^®^ RG 503, 50:50 (d,l-lactide/glycolide), obtained as amorphous powder (a kind gift from Roland Klein, Ernst-Berl-Institute for Chemical Engineering and Macromolecular Chemistry, Darmstadt University of Technology, Darmstadt, Germany). Its principle characteristics are molar mass range of 24,000–38,000 g/mol, inherent viscosity of 0.32–0.44 dL/g, and glass-transition temperature of 44–48 °C, as determined by the manufacturer (Boehringer Ingelheim, Ingelheim, Germany). Analytical grade dichloromethane (DCM) was from VWR International (Darmstadt, Germany). The 4-HR (98%) from Aldrich (Steinheim, Germany) was used without further purification (Table 1).

### 3.2. Film Preparation

PLGA–4-HR composite films were developed by solvent casting. In 25 mL glass jars with screw top, 0.5 g of PLGA and varying amounts of 4-HR (0, 15, 30, 50, 75, 150, 250 mg) were dispersed in 7.5 mL DCM, respectively, and magnetically stirred for 1 h until total dissolution. The prepared solutions were allowed to stand undisturbed for 30 min eliminating gaseous bubbles, and 200 µL clear samples were cast onto levelled round glass coverslips of 15 mm diameter (No. 1, VWR International), pre-cleaned in acetone. DCM was allowed to evaporate slowly in air for 4 h at 8 °C in a cooling incubator (tritec KB 8400, tritec-Gesellschaft für Labortechnik und Umweltsimulation, Hannover, Germany) to prevent the formation of bubbles, followed by evaporation under high vacuum in a desiccator over 10 Å molecular sieves (Carl Roth, Karlsruhe, Germany) for 16 h. The coated samples were further oven-dried at 40 °C for 3 h to remove traces of remaining solvent that could act as biocide [49], before they were sealed and stored light-protected at 4 °C until further characterisation and analysis. The amount of pure 4-HR in PLGA-composite film discs was calculated to be 0.03, 0.06, 0.1, 0.15, 0.3, and 0.5 mg/mg PLGA or 0.2, 0.4, 0.7, 1.1, 2.2, and 3.7 mg/cm^2^ surface area of film, respectively.

### 3.3. Film Thickness and Density

The thickness of film specimen, coated and uncoated, was measured using a handheld micrometer (Mitutoyo dial thickness gauge 7301, Mitutoyo Deutschland, Neuss, Germany) with 0.01 mm accuracy and expressed as an average of seven random measurements and standard deviation. Six replicates of sample mass were determined with an analytical balance accurate to 0.1 mg (Explorer Pro EP214, Ohaus Corporation, Pine Brook, NJ, USA) and the mass values were averaged. Coating mass and thickness of each PLGA film were calculated by subtracting the corresponding values from coated specimen and uncoated coverslips, respectively. The density ρ of each film was evaluated according to the following equation:

(1) $$\rho =\frac{m}{A\;\cdot \;d}$$

where *m* is the coating mass, *d* is the film thickness, and *A* is the given film area of 1.77 cm^2^.

### 3.4. Microorganisms and Inoculum Preparation

Microbial strains used as indicator organisms and corresponding incubation conditions are reported in Table 4.

The microbial stock cultures were stored frozen in liquid growth medium at −80 °C with glycerol at a final concentration of 15% (*v*/*v*) until required. Spore suspensions of the filamentous fungi were prepared by suspending conidia from 7- to 10-day-old cultures in 10 mL 0.05% (*v*/*v*) Triton X-100 (Carl Roth) and were stored at 4 °C [50]. For recovery of bacterial and yeast strains, one loop from each stock suspension was streaked on appropriate agar plates and incubated for a period sufficient to obtain single colonies. Microbial cells and spores were enumerated with three replicates by a plate-count method for viable cells. Serial dilutions of each cell or spore suspension were plated by surface spreading onto suitable agar medium and the number of visible colonies reported as colony-forming units per milliliter (CFU/mL). Each microbial culture was adjusted to 1 OD_600_ unit with a standard curve relating optical densities at 600 nm (OD_600_) to viable cell numbers [51]. This allowed the standardisation of assay inoculums by OD_600_ measurements with an Ultrospec 2100 pro spectrophotometer (Amersham Biosciences, Uppsala, Sweden).

### 3.5. Antimicrobial Activity Screening by Agar Overlay Assay

**Figure 2:**
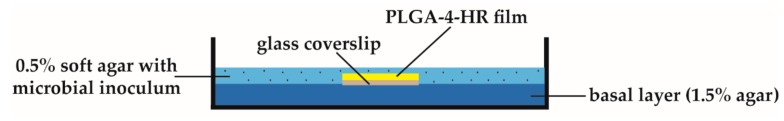
Schematic representation of the agar overlay assay.

Based on the Kirby–Bauer disc susceptibility test [29], the antimicrobial activity of 4-HR in PLGA composites was evaluated by determining the zone of growth inhibition using an agar overlay assay. This method was adapted from a seeded-lawn overlay spot assay [52] and adjusted to supported discs of thin polymer films as biocide carriers. The method described was set up to develop a bilayer system (Figure 2), which could provide a reproducible and accurate assay to quantitate antimicrobial susceptibility of drug releasing polymers.

For the basal layer, Mueller–Hinton broth (MHB) or Sabouraud 2% dextrose broth (SDB) agar plates were prepared by pouring approximately 30 mL of sterilized plating medium (Table 4) with 1.5% (*w*/*v*) agar into Petri dishes to a depth of 5–6 mm and allowed to solidify. The described PLGA-4-HR film discs were disinfected by shaking in 70% (*v*/*v*) aqueous ethanol for 10 min and dried in a sterile atmosphere for 2 h at 37 °C. Films with neat PLGA represented the negative control. For agar diffusion, each film disc was placed onto the surface of the basal layer and overlaid with 20 mL seeded soft agar (0.5% *w*/*v* agar in plating medium) pre-warmed to 42 °C. The final inoculum size for soft agar was 1 × 10^6^ CFU/mL of bacteria and *P. italicum*, 2.5 × 10^5^ CFU/mL of yeasts, and 1 × 10^5^ CFU/mL of *B. cinerea*, respectively. After solidification of the top agar, incubation of the plates occurred at strain-specific temperatures (Table 4) for 22 h, except for fungal cultures requiring incubation periods of 48 h. The agar overlay assays were independently repeated three times using different batches of active agents and microbial suspensions on different days.

The inhibition size around the film discs (colony-free diameter) was determined with a Haloes Caliper accurate to 0.1 mm (IUL Instruments, Königswinter, Germany). Each inhibition halo was expressed as an average of at least six measurements at random perimeter locations. All measurements were done by the same investigator to eliminate inter-person bias.

### 3.6. Determination of MIC Values

Following disc diffusion, the MIC values were calculated according to two different mathematical models based on free and dissipative diffusion [32]. Plots of 4-HR concentration versus the respective diameters of inhibition zones for individual discs were fitted to Equations (2) and (3) as the development of an inhibition zone depends on the nature of diffusion of 4-HR through the agar (Figure 2). In the free diffusion model, the value of MIC was determined as the zero intercept of a linear regression of the squared size of these inhibition zones, x, plotted against the natural logarithm of 4-HR content per disc, *c*:

(2) $$\ln\left(\mathrm{MIC}\right)=\ln\left(c\right)-\;\frac{x^{2}}{4Dt}$$

where *D* is the diffusion coefficient, presumed to be independent of concentration, and *t* is time of 4-HR diffusion. According to the dissipative diffusion model, the value of MIC was determined using a plot where the size of inhibition zone, x, plotted against the natural logarithm of 4-HR concentration, *c* is expressed as:

(3) $$\ln\left(\mathrm{MIC}\right)=\ln\left(c\right)-2D^{-1}\left(V\;\pm \;\sqrt{V^{2}-4D}\right)\;x$$

In addition to the above denoted diffusion coefficient *D*, *V* is a coefficient characterising the dissipation rate. Since 4-HR was dispersed in solid PLGA films, all MIC values were expressed in mass fractions (% (*w*/*w*)) of total 4-HR ingredient.

### 3.7. Statistical Analysis

Multiple replications of measurement with mean and standard deviation of mean were applied throughout the experiments. Linear regression using a least-squares algorithm was carried out with MS Excel 2010. Significance of data on film thickness and density was determined by one-way analysis of variance (ANOVA) with pairwise comparisons using post hoc Tukey’s HSD (honestly significant difference) test [53]. The values were considered significantly different at *p* < 0.05. The photograph of agar overlay assay and the plots showed in figures were only the representative.

## 4. Conclusions

Supported PLGA films with 4-HR as active agent in concentrations up to 3.7 mg/cm^2^ surface area were successfully prepared by a solvent casting technique and applied in a modified seeded-lawn agar overlay assay. To the best of our knowledge, this is the first report on a quantitative assay performed directly with different bacterial, yeast, and fungal indicator strains to provide reproducible MIC values of 4-HR-loaded PLGA films. Our results suggest that incorporation of 4-HR into biocompatible PLGA polymer matrices could produce powerful composite films for inhibition and transmission of microbial growth. The proprietary biomaterial opens up new perspectives in health, pharmaceutical, biomedical, and packaging applications.

## Figures and Tables

**Table 1:** Physico-chemical characterisations of 4-hexylresorcinol (4-HR).

Chemical Structure	Physico-Chemical Properties [26]
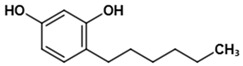	Molar mass 194.27 g/mol Melting point 62–67 °C Log *P*_o/w_ 3.88

*P*_o/w_: octanol-water partition coefficient.

**Table 2:** Effect of 4-HR on thickness and density of poly(lactic-co-glycolic acid) (PLGA) films.

4-HR Content (mg/mg PLGA)	Thickness (µm)	Density (g/cm^3^)
Neat PLGA	75 ± 18 ^a^	0.94 ± 0.06 ^a,b^
0.03	81 ± 7 ^a^	0.83 ± 0.12 ^a^
0.06	83 ± 19 ^a^	0.81 ± 0.07 ^a^
0.1	66 ± 12 ^a^	0.99 ± 0.08 ^a,b^
0.15	64 ± 14 ^a^	1.10 ± 0.17 ^b^
0.3	67 ± 8 ^a^	1.06 ± 0.13 ^b^
0.5	92 ± 15 ^a^	1.08 ± 0.10 ^b^

Mean values ± standard deviation. Different superscript letters in the same column represent significant differences (^a,b^ *p* < 0.05). Groups with dual letters (^a,b^) are not statistically different from other groups marked with a or b.

**Table 3:** Minimum inhibitory concentrations (MIC values expressed in % (*w*/*w*)) and corresponding coefficients of determination (*R*^2^) of 4-HR-loaded PLGA films against various indicator strains.

Indicator Strains	Free Diffusion		Dissipative Diffusion	
	MIC *	*R*^2^ *	MIC *	*R*^2^ *
Gram-positive bacteria				
*Bacillus pumilus* DSM 361	8.3	0.995	2.6	0.987
*Kocuria rhizophila* DSM 348	8.6	0.967	3.5	0.96
*Listeria innocua* DSM 20649	6	0.978	1.5	0.966
*Staphylococcus carnosus* DSM 20501	3	0.998	0.4	0.992
Gram-negative bacteria				
*Aeromonas ichthiosmia* DSM 6393	7.5	0.993	2.4	0.988
*Escherichia coli* ATCC 10798	6	0.987	1.2	0.975
*Pseudomonas putida* DSM 291	28.5	0.999	12	1
*Raoultella planticola* DSM 3069	9	0.995	2.3	0.994
Yeasts				
*Rhodotorula mucilaginosa* DSM 70825	7.5	0.982	3	0.975
*Yarrowia lipolytica* DSM 70561	7.5	0.977	3	0.972
Filamentous fungi				
*Botrytis cinerea* DSM 4709	6	0.992	0.8	0.989
*Penicillium italicum* DSM 2754	9	0.999	3	0.995

* Average MIC and *R*^2^ values from three repeats are presented.

**Table 4:** Indicator strains used in this study.

Indicator Strains ^a^	Liquid Growth Medium ^b^	Plating Medium ^b^	T (°C)	Viable Cell Count (CFU/mL) ^c^
Gram-positive bacteria				
*B. pumilus* DSM 361	NB	MHB	28	3 × 10^8^
*K. rhizophila* DSM 348	NB	MHB	28	7 × 10^7^
*L. innocua* DSM 20649	BHI	MHB	37	9 × 10^8^
*S. carnosus* DSM 20501	TSB	MHB	37	3 × 10^8^
Gram-negative bacteria				
*A. ichthiosmia* DSM 6393	NB	MHB	28	4 × 10^8^
*E. coli* ATCC 10798	TB	MHB	37	8 × 10^8^
*P. putida* DSM 291	NB	MHB	28	8 × 10^7^
*R. planticola* DSM 3069	NB	MHB	28	2 × 10^8^
Yeasts				
*R. mucilaginosa* DSM 70825	YM	SDB	28	2 × 10^7^
*Y. lipolytica* DSM 70561	YPD	SDB	28	1 × 10^7^
Filamentous fungi				
*B. cinerea* DSM 4709	MEP	SDB	28	1 × 10^6 d^
*P. italicum* DSM 2754	MEP	SDB	28	4 × 10^8 d^

^a^ ATCC, American Type Culture Collection (LGC Standards, Wesel Germany); DSM, Deutsche Sammlung von Mikroorganismen (Leibniz Institute DSMZ, Braunschweig, Germany); ^b^ BHI, brain heart infusion broth; MEP, malt extract peptone broth; MHB, Mueller–Hinton broth; NB, nutrient broth; SDB, Sabouraud 2% dextrose broth; TB, tryptone broth; TSB, tryptic soy broth; YM, universal yeast medium; YPD, yeast extract peptone dextrose broth; delivered from Merck (Darmstadt, Germany) and Roth (Karlsruhe, Germany); ^c^ For cultures growing to an OD_600_ of ~1; ^d^ Spore concentrations.
